# Perceptions of cancer risk communication in individuals with overweight or obesity– a qualitative interview study

**DOI:** 10.1186/s12889-025-23056-w

**Published:** 2025-05-23

**Authors:** Åsa Grauman, Erica Sundell, Jessica Nihlén Fahlquist, Mariann Hedström

**Affiliations:** 1 Centre for Research Ethics and Bioethics, Uppsala University, Box 564, Uppsala, SE-751 22 Sweden; 2 Department of Public Health and Caring Sciences, Uppsala University, Box 564, Uppsala, SE-751 22 Sweden

**Keywords:** Prevention, Cancer, Obesity, Overweight, Risk communication, Qualitative research, stigma

## Abstract

**Background:**

Addressing obesity and overweight is crucial for cancer prevention. However, ensuring that such efforts do not harm individuals with obesity requires careful consideration to avoid reinforcing the widespread stigmatisation of individuals with obesity. This study aims to explore how individuals who have overweight or obesity perceive cancer risk information addressing obesity as a cancer risk factor.

**Methods:**

Semi-structured interviews were conducted during autumn 2023 and spring 2024, with 11 Swedish men and women, aged 24 to 70 years, who self-assessed as having overweight or obesity. The collected data were analysed using reflexive thematic analysis as described by Braun and Clarke.

**Results:**

The first theme, Reflecting on personal risk, included the subthemes: It is about me and my body and Awareness can be a burden. The second theme, Healthcare encounters: building trust and providing support, included the subthemes: Past encounters influence how risk information is perceived, Need to act and succeed, and Consider receptivity and power imbalances. The third theme, Distribution of responsibility and blameworthiness, included subthemes: Being personally blamed and fearing increased discrimination and A need for shared responsibility.

**Conclusion:**

Participants experienced that risk information, when presented as simplified associations between obesity and cancer, felt personally relevant but difficult to act upon, and therefore burdensome to bear. Consequently, the information risks failing to prevent cancer and may instead cause harm to the people it purports to benefit. Participants suggested several ways to improve the provision and benefit of such information, including introducing multiple ways to act to reduce cancer risk that goes beyond weight reduction, and raising public awareness of the causes of obesity. Efforts to communicate risk, both to the public and to patients, require better ethical considerations of their benefits and harms. Furthermore, risk communicators should act with compassion and responsibility.

**Supplementary Information:**

The online version contains supplementary material available at 10.1186/s12889-025-23056-w.

## Introduction

Cancer prevention focuses on modifiable risk factors, which account for 30–50% of all cancer cases [1]. The European Code Against Cancer (ECAC) present twelve actions individuals can take to help prevent cancer. One of them is maintaining a healthy weight [2]. Obesity accounts for approximately 17% of the total cancer mortality [3] and increases the risk of several types of cancer, [1] including post-menopausal breast cancer (BC) [4] and colorectal cancer (CRC) [5]. Obesity is a chronic disease with a complex aetiology [6] that has more than doubled worldwide since 1990. Currently, about 43% of the adult population worldwide have overweight, and 16% have obesity [7]. In Sweden, approximately 50% of the adult population have either overweight or obesity [8].

Awareness of obesity and overweight as cancer risk factors has been low among healthcare professionals (HCPs) and laypeople, [9, 10, 11] and the lowest among individuals with low socioeconomic status [10, 12]. Meanwhile, obesity is overrepresented among socially disadvantaged groups [13]. Awareness of cancer risk factors is needed for making informed choices and is associated with a greater willingness to participate in CRC screening [12].

Cancer prevention in primary healthcare has long focused on smoking, [14] while rarely mentioning obesity [9]. Since 2011, Sweden has implemented national disease prevention guidelines to address patients’ unhealthy lifestyles in clinical practice. Patients who have overweight or obesity are identified as one of several high-risk groups that should be prioritised [14]. A Swedish report found that many HCPs perceive lifestyle discussions as a sensitive topic, with weight and cancer being the most sensitive issues due to fears of intruding on patients’ privacy [15].

When addressing obesity as a risk factor for cancer, it is important to consider the risk of reinforcing the widespread stigmatisation of individuals with obesity [16]. Weight stigma is defined as ‘negative attitudes towards, and beliefs about others because of their weight, manifested by prejudices towards people who have overweight and obesity’, [17] and is reinforced by perceptions that behavioural causes are under high personal control [18]. Discrimination and offensive behaviour against individuals with obesity takes place across all sectors of society, leading to inequalities in health, education and economy [3, 6]. Negative attitudes among healthcare professionals towards individuals with obesity can influence their interactions with patients [19]. Individuals with obesity have reported experiences of discrimination, including derogatory comments [20] and the attribution of all health issues to excess weight [16, 21, 22]. These experiences can lead to feelings of shame, mental distress, body image dissatisfaction and the escalation of unhealthy behaviours [20, 22, 23]. Weight stigma can also cause mistrust of doctors, anticipation of discrimination and avoidance of healthcare [22, 24].

Cancer is, in itself, a stigmatised disease due to negative perceptions that evoke feelings of dread [25, 26, 27]. The attribution of cancer causes to high personal responsibility increases the stigma, [26, 28, 29] and even more if the behaviour is perceived as unacceptable by society. This can lead to patients feeling blamed for their disease and fearing being denied access to care [30]. Linking obesity to cancer can, therefore, have negative consequences for cancer patients. Swedish BC and CRC patients struggling with overweight have reported experiencing a double burden, as they must not only fight cancer but also contend with insensitive comments about their weight [31].

Previous research has focused on the stigmatisation of individuals who have obesity or overweight, but not specifically within the context of cancer risk communication. Addressing obesity as a cancer risk factor is crucial for cancer prevention. However, ensuring that such efforts do not harm individuals with obesity requires precaution and careful consideration. This study aims to explore how individuals with overweight or obesity perceive cancer risk information that identifies obesity as a cancer risk factor.

## Methodology

The study employed an exploratory qualitative design.

### Participants

We reached out to a Swedish patient organisation for individuals with overweight and obesity (Riksförbundet Obesitas Sverige) to recruit eligible participants. Using convenience sampling, the organisation distributed an invitation to participate in the study to its members. Inclusion criteria were: aged 18 or above, have overweight or obesity, and able to read and speak Swedish. The exclusion criterion was having a history of cancer. The participants self-assessed whether they met the inclusion criteria of having overweight or obesity. Participants were offered a gift certificate worth 150SEK (≈ 13 €) as a token of appreciation. Eight individuals willing to participate contacted the first author, ÅG. However, two decided not to participate, resulting in six participants being recruited. To recruit more participants, we approached the Centre for Obesity, a specialist unit in Stockholm that provides obesity treatment. They posted information about the study in their waiting room and on their website. Three additional individuals signed up and were recruited. Two additional participants were recruited through snowballing. Participants’ characteristics are presented in Table 1.

**Table 1:** Participants’ characteristics

	*N*	Median, range
Total	11	
Sex		
*Female*	8	
*Male*	3	
Age		37, 24–70
Born in Sweden (yes)	10	
Educational level		
*Primary school*	0	
*Secondary school*	6	
*University*,* post-secondary education*	5	
Training as a health professional		
*No*	8	
*Yes*	3 (1 registered nurse, 2 nurse assistants)	

### Data collection and analysis

The individual interviews were conducted during autumn 2023 and spring 2024 by ÅG, using a semi-structured interview guide (Supplementary file) covering questions about participants’ experiences with, attitudes towards and reactions to cancer risk information. Similar questions have previously been used with a patient population [31]. Risk information included all sources that participants encounter in their daily lives, such as newspapers, websites and healthcare visits. At the beginning of each interview, participants were also asked background questions (Table 1). Seven interviews were conducted online (video call through Zoom), two were conducted face-to-face, and two over the phone. The interviews, which lasted between 21 and 44 min (mean 36 min), were audio-taped and transcribed verbatim by a professional transcription company. All personal identifiers were removed from the transcripts to ensure anonymity.

The data were analysed inductively using reflexive thematic analysis (RTA), according to Braun and Clarke [32, 33]. The analysis started with familiarisation with the data by repeatedly reading the transcripts. In the next step, all co-authors read and coded the same interview individually and then met to discuss the coding, aiming to enhance understanding rather than to reach a consensus. ÅG and ES completed the coding, and ÅG continued by sorting the codes into potential themes. During this iterative process, themes were either collapsed or broken down, while continuously going back and forth between transcripts, codes and themes. The final set of themes and subthemes were discussed among the researchers, who labelled them with a brief description to capture its essence [32]. The software Atlas.ti 9 was used to organise the codes and initial themes.

Thematic analysis was chosen for its recognition of the researchers’ subjectivity as a valuable resource in the analysis process. Themes are therefore seen as the result of the researchers’ active choices [33]. We adopted a perspective in which the exploration of subjective experiences does not aim to uncover an objective truth, but recognises that both the participants’ experiences and the researchers’ interpretations are shaped by cognition, language, and social constructions. The research team comprised women with academic backgrounds in public health, nursing, and ethics, all with extensive experience in qualitative research. The data were reflected upon through the researchers’ pre-understandings of risk perception and stigmatisation.

## Results

Three themes and eight subthemes were identified (Table 2).

**Table 2:** Themes and subthemes

Themes	Reflections on personal risk	Healthcare encounters: Building trust and providing support	Distribution of responsibility and blameworthiness
Subthemes	It is about me and my body	Past encounters influence how risk information is perceived	Being personally blamed and fearing increased discrimination
	Awareness can be a burden	Need to act and succeed	A need for shared responsibility
		Consider receptivity and power imbalances	

### Reflections on personal risk

Participants generally reported having limited knowledge about cancer and its risk factors, and most had not heard about the connection between overweight and cancer before the interview. The few who were familiar with the link had learned about it through a physician, nursing school or non-healthcare-related sources. All participants were well aware of other health risks associated with having overweight and were, therefore, not surprised to hear about the association between having overweight and cancer. Several also expressed a curiosity about learning more about how cancer develops and what increases the risk of cancer.

#### It is about me and my body

Some participants expressed that the information about overweight increasing the risk of cancer was highly important to them, as they constitute the high-risk group. The information felt personal because it was about them and their bodies.

*So it’s good that we bring up these things and reconsider them. And as you say*,* when it’s personal*,* it still becomes like… maybe it was more serious like that. Because it’s still about me*,* my life. (Participant 2*,* male)*

Some of the older participants expressed that they wished they had received this information much earlier in life, noting that it would be awful to develop cancer and only then learn about the increased risk. Participants reflected that weight is often perceived as being about physical appearance when one is young. However, as they grew older, health became more important, both for their own well-being and to be able to support their families. Similarly, one younger participant was positive towards weight loss messages that focus on health rather than physical appearance.

*That you need to reflect on your lifestyle*,* how you have ended up in this situation where you have an increased risk of cancer and are unaware of it. It would have been much nicer to have been told much earlier what you need to change*,* instead of*,* in the worst case*,* having cancer and could have avoided it but not done anything about it. (Participant 5*,* female)*

#### Awareness can be a burden

Being identified as belonging to a high-risk group for cancer felt tough. As having overweight is also a risk factor for many other diseases, one woman described herself as ‘a walking risk factor’. For most participants, cancer was perceived as a scary disease, making weight loss feel especially important. However, there were exceptions among those who had experienced cancer within their families. For these individuals, cancer had become normalised and was viewed as any other disease, leading to reduced fear. All participants acknowledged that no one chooses to have overweight, and that they had repeatedly tried to lose weight in the past. Therefore, they thought that receiving risk information without being offered possibilities to influence their weight could be counterproductive, and lead to excessive eating, anxiety, hopelessness and reinforcing their identity as a person who has overweight.

*It is obvious that it is not pleasant to hear*,* that there is research that shows that it is so. In some way*,* you already feel like you… you are perhaps a bit more exposed to that because you are overweight right from the start. But that it would mean getting some diseases*,* I have not understood that before. […] I kind of felt that*,* now*,* I probably have to deal with this*,* with my weight or try to lose weight. But I also know how difficult it is. My body wants nothing more than to be the size it has been for several years now. (Participant 3*,* female)*

In contrast, some participants stated that they would not react to the information about the association between obesity and cancer. Some expressed that cancer is so common in society that ‘no one is spared in the end’. Others perceived that ‘everything causes cancer’, and that thin individuals can get cancer too, suggesting that having overweight might not be as dangerous as it is often portrayed. Other reasons for their lack of reaction were that they already knew about the fact or felt sceptical towards science communication in general.

### Healthcare encounters: Building trust and providing support

Participants discussed how previous healthcare encounters influenced their trust in HCPs and shaped how they perceived information from them. They also emphasised that risk information needs to be accompanied by adequate support.

#### Past encounters influence how risk information is perceived

Participants shared negative experiences during healthcare visits. For instance, HCPs had sometimes given irrelevant lifestyle advice without first assessing the participants’ actual lifestyles, and weight was often judged as the sole cause of all health problems. Participants raised the importance of trusting the source of information, regardless if it is information to the public or individual. However, due to past negative encounters with or discrimination from HCPs, some participants had lost trust and did not want to receive information from physicians.

*IF: Yes*,* I would probably dismiss that [information about increased cancer risk from the HCP]. I wouldn’t take that as very credible. […] I mean*,* I don’t trust healthcare in terms of treatments and such. If I’m going to see a doctor*,* it should be someone I feel I can trust. (Participant 3*,* female)*

One woman shared the insensitive manner in which a physician informed her that having overweight increased her risk of colorectal cancer. She described experiencing that the physician was looking at her as if she was disgusting, which made her feel sad.

*When I started talking about this*,* that I wanted to do a gastric bypass*,* there was a doctor at the health centre who said*,* “yes*,* it’s good because then you can avoid cancer in the future”. And then whether it was right or wrong*,* I don’t know. But he actually said that*,* yes*,* that’s good*,* well*,* then you can avoid bowel cancer. […] I felt sad inside. But maybe he felt it was his duty to enlighten me. I don’t know. Some doctors are very… they can disconnect*,* they have a kind of autopilot sometimes when they talk to people*,* so it becomes almost a bit methodical and cold. But they may have to do so in order to cope with their profession […]*.

*Interviewer: Was it the way he said it or the message of what he said…*.

*It was both the way and the message*,* because he looked at me… Because I thought I felt that he only saw me as being disgusting. That’s how it felt. You can carry a lot inside when you are large because it becomes like a fortress in the end. […] I would have liked him to have refrained from saying anything at all. Because it went so wrong. It felt very wrong anyway. (Participant 6*,* female)*

Participants expressed a desire for HCPs to make an effort to understand them as individuals, explore the underlying causes of their overweight and address their individual needs.

#### Need to act and succeed

Most participants had been advised by HCPs to lose weight, but few were offered support or information about treatment options. Since they were already aware of the need to lose weight, this advice without support felt useless and, at times, offensive, as though they were being labelled as stupid.

Some participants were currently undergoing or on the waiting list for weight loss interventions, which they perceived made it easier to deal with risk information. However, several participants expressed dissatisfaction with the support provided, experiencing that HCPs often focused on unattainable and rigid weight goals, as well as strict recommendations for physical activity and calorie intake, leaving little room for success. Furthermore, some expressed a lack of empathy for their struggles and acknowledgement for their achievements. This added to the feelings of failure and self-hate towards their bodies, feelings that many had carried throughout their lives. Participants suggested a re-directed focus on overall well-being and the promotion of other actions to decrease the risk of cancer. They emphasised the importance, as a high-risk person, of taking preventive actions, such as being mindful of cancer symptoms and attending health check-ups and screening programmes. In that way, the cancer risk and overall health could be improved without requiring the attainment of strict weight goals.

*I am the one who has failed*,* and that arouses a lot of negative feelings. And that maybe that’s it*,* you want to get away from… or that I had wanted to get away from the negative feelings*,* and that more*,* but what can you do to be healthy*,* that the goal doesn’t have to be that*,* now I’m going to lose weight*,* because it can also be a trigger*,* because it also becomes very easy to fail*,* and instead of seeing it as a way to succeed or fail*,* but how can I live as well as possible with my circumstances. (Participant 10*,* female)*

#### Consider receptivity and power imbalances

Participants stated that the individual needs to be receptive to information, which is often the case when the individual actively seeks out information or support. They emphasised the importance of trustworthy and understandable information readily available on public websites and in settings such as waiting rooms of primary healthcare centres. Similarly, they felt that seeking information on your own, as opposed to being told, improves the perceived sense of control and empowerment. However, they recognised that personal encounters with HCPs have the benefit of allowing for follow-up questions. Some participants had positive experiences of receiving weight-related information in a group with others in a similar situation or through patient organisations, and saw it as a way to reduce the power imbalances.

Participants noted that it is not always appropriate to inform individuals about the increased risk of cancer associated with having overweight, as it is often linked to feelings of shame and guilt. They emphasised the need for HCPs to be empathetic and sensitive to potential negative reactions, considering the mental state of their patients. If a person is having a bad day or experiencing mental health issues, information about how having overweight increases the risk of cancer may be overwhelming.

*It’s good to know that it increases the risk*,* but if you’re going to give that information*,* above all to a single individual like this… it’s good to sort of get a feel for the room; will this person… does it help this particular person now*,* regarding why he or she is here*,* or will it just create anxiety and the person can do nothing about it. (Participant 10*,* female)*

On the contrary, one participant did not appreciate the unease shown by HCPs in addressing weight. The participant stated that since everyone is aware of their weight, there is no need to tiptoe around the issue.

### Distribution of responsibility and blameworthiness

Participants described how individuals who have overweight or obesity are personally blamed for their weight by society. They urged risk communicators to take responsibility for avoiding the reinforcement of such public perceptions.

#### Being personally blamed and fearing increased discrimination

The participants placed cancer risk information within the context of society’s norms about weight: ‘being overweight is wrong and being thin is right’. They stated that society tends to blame individuals for having overweight, attributing it to personality traits such as being lazy. However, they felt that the complexity and different causes of obesity are not adequately acknowledged in society. Some felt that having overweight receives more attention and judgement compared to other risk factors. They expressed that, as individuals with overweight are already oppressed in society, the additional burden of risk information felt like a double burden to bear. Some raised concerns that if awareness of the association between having overweight and cancer increased, individuals would be blamed for their cancer diagnosis. Similarly, one participant voiced concerns about potential future discrimination, such as being denied medical care due to their increased risk. Others, however, had difficulties imagining such consequences.

*I think it’s also sort of more like this culturally…that it’s sort of fed in that if you’re fat like this*,* it’s sort of your own fault and to not*,* sort of*,* do something about it*,* it’s like you’re weak or bad or something. And then I know that there is research that it’s not like that*,* but that it can be an eating disorder and there are other reasons. (Participant 10*,* female)*

On the other hand, some participants thought that raising awareness about the increased cancer risk associated with obesity could shift positive attention towards recognising obesity as a serious health issue instead of a beauty issue. Thereby, it could help reduce the stigma attached to weight.

#### A need for shared responsibility

The participants addressed the issue of responsibility related to risk management. While they acknowledged that individuals have a responsibility in maintaining their own health, many felt that the causes and treatments of obesity often lie beyond their own control. The responsibility, therefore, needs to be shared with other stakeholders in society. Many participants thought HCPs have the responsibility to inform patients about risks, provide treatment options, guide them on where to find more information and ensure access to adequate treatment.

One participant highlighted the responsibility of researchers and thought they should be active in the public debate by taking responsibility for how research results are disseminated, correct false citations and errors, and object to stigmatising headings.

*In much of the media communication that takes place around health*,* I think there is a risk that it will either lead to blame*,* or that it can lead to negative feelings… So*,* I think*,* it is very important that you as a researcher or institution are very active in how that information is disseminated. You can’t influence how newspapers or Instagram accounts set their headlines or wording… but you have to be active in the work to respond to it*,* and follow up on the interpretation of your research results. (Participant 9*,* male)*

Other participants thought that experts have a responsibility to present nuanced risk messages, be cautious when communicating risks, and only communicate the ‘really important and certain risks’; otherwise, it may appear that everything causes cancer and people will stop listening. They also raised the importance of educating people about the complexity and multiple causes of obesity, e.g., eating disorders, medication use and mental illness.

*Because you think it’s sort of self-selected and don’t see that this person might have an underlying illness. So*,* I think a lot of understanding is required for increased knowledge to be a positive thing in how larger people are treated by others. I think*,* then you also have to go out with information so that people think okay*,* if someone is overweight*,* what factors could this be due to? And then being obese increases the risk of this and that. So*,* you kind of have the whole chain; why are you overweight? There are a lot of reasons. (Participant 7*,* female)*

Many participants also highlighted the need for political action to address the rising prevalence of obesity in society, particularly reflecting on the increase in childhood obesity as a societal issue. One woman mentioned that gym memberships are often expensive and not accessible to everyone. Instead, she advocated for outdoor gyms and for the organisation of support groups at local primary healthcare centres.

## Discussion

In this study, individuals with overweight or obesity were interviewed about their experiences and perceptions of information about how overweight and obesity increase the risk of cancer. Participants related the topic to their experiences of being informed about other health risks and the pervasive societal message that having overweight is “bad”, although cancer was generally perceived as more serious. Hence, cancer risk information cannot be separated from past experiences and societal norms.

In the first theme, Reflections on personal risk, most participants stated that they were unaware of overweight/obesity being a risk factor for cancer. Being identified as a high-risk individual felt challenging and added to the burden of other health risks associated with having overweight/obesity. Conversely, some felt a bit sceptical about the association between overweight and cancer, pointing out that thin people get cancer, too. Referring to atypical cases is a common tendency, which can introduce doubts about the influence of specific risk factors [34]. Risk communicators, therefore, face the challenge of designing information that neither frightens nor is dismissed by the audience.

The information about the increased cancer risk also heightened the perceived importance of weight loss. However, the participants were not optimistic about succeeding in the weight loss efforts, which could lead to feelings of hopelessness and worry. This also relates to the second theme, Healthcare encounters: building trust and providing support, where participants reported that recommendations from HCPs to lose weight were often pointless without adequate support, as they were already well aware of their need to lose weight. The importance of being able to act on risk information in order to benefit from it has been raised by both the public and patients [31, 34]. The ability to act on risk information relates to empowerment, which has been suggested as a positive outcome of risk information, conceptualised as a sense of control over the risk [35]. It includes several key aspects: knowledge of the risk factor and its outcomes, the ability to take actions to influence the risk, and how to use the healthcare systems effectively. Empowerment also includes being able to manage one’s feelings and maintaining hope for the future [35].

As previous research has shown, [16, 20, 22, 24] participants expressed experiencing both internalised stigma, in terms of self-hatred, and external stigma, in terms of negative encounters with HCPs that eroded their trust in the healthcare system. Participants perceived a lack of empathy and understanding for their situation during weight loss interventions, particularly due to the unrealistic goals set by HCPs. Given that individuals may not be able to reach exact weight goals, at least not immediately, HCPs should consider presenting other feasible actions to prevent cancer. This approach could help reduce the sense of failure experienced by some patients. For instance, HCPs could promote the remaining eleven recommendations of the ECAC, such as promoting vaccines, screening, and physical activity [2].

Participants also asked for a more person-centred approach, which previous studies also proposed [36, 37]. Moreover, guidelines for disease prevention state that HCPs should provide person-centred counselling when assessing the lifestyles of patients with overweight/obesity. These guidelines also state that patients should be ‘treated and cared for with respect and consideration and perceive the treatment as respectful, competent and empathetic’ [38]. A Norwegian study discussed the lack of person-centred care in weight management, attributing this to a negative prioritisation of standardised care, which emphasises standardised questions, treatments and goals [37]. Similarly, Swedish primary healthcare physicians and nurses report a lack of counselling skills, referral options, and sufficient time to listen to patients as obstacles to implementing the guidelines [14]. However, if guidelines are followed without empathy, a person-centred approach, or adequate support, there is a risk of causing more harm than good. Therefore, it might be better to refrain completely from addressing patients’ weight if it cannot be done with the necessary quality and care.

In the third theme, Distribution of responsibility and blameworthiness, participants addressed the negative societal perceptions surrounding personal blame for having overweight. They noted that society often judges people for having overweight more harshly than for other risk factors. Learning about the increased cancer risk associated with having overweight becomes an additional burden, echoing sentiments previously expressed by cancer patients who have overweight [31].

While participants acknowledged their own role in weight management, they also emphasised the need to share the responsibility with others. Several raised the need for more responsible public information and were unsatisfied with current risk communication, which often relies on simple statements about established associations. Instead, participants thought that experts and institutions should provide more nuanced messages and educate the public about the complex causes of obesity to debunk negative perceptions of personal characteristics as the causes of obesity. Risk information to the public also needs to enhance recipients’ perceived control by providing clear guidance on how to reduce the risk. Furthermore, one male participant expressed that institutions and experts bear responsibility for how their information is disseminated and interpreted by others. He suggested they should take a more active role in the public debate, for example, by correcting misinterpretations and challenging misleading headlines.

Risk communication to the public should be based on principles of ethics [39]. However, such efforts often lack clear objectives, [39] and ethics considerations are often limited to the obligation of transparency (in this case, informing the public about the increased cancer risk associated with having overweight). It is possible that institutions/experts/researchers do not perceive their communication as a form of public health intervention. As a result, they may underestimate the impact of their communication and their power position. Furthermore, routines for assessing the ethical aspects in public health interventions are not implemented in the same way as in clinical interventions. Kass has developed a framework for the systematic ethical analysis of public health programmes. The framework comprises six key questions: (I) What are the primary objectives? (II) What is the evidence on effectiveness? (III) What are the potential burdens? (IV) What are the possibilities to reduce the burden or choose alternative methods? (V) Is the distribution of benefits and burdens fair? (VI) Are the burdens acceptable in relation to the benefits? [40] As a complement to Kass’s consequentialism framework, virtue ethics can also be applied to public health, adding important values. Arguably, public health professionals should be attentive, compassionate and attempt to understand the perspectives of those receiving health messages. Ideally, they should recognise that public health does not merely involve conveying facts about what is good for the health of the majority, but also considering individuals, their values and their lived experiences. For example, a virtue ethical public health professional would consider the risk of stigmatisation and the different perceptions of quality of life as important factors when communicating lifestyle-related risks [41].

Finally, participants also reflected on changes in society that contribute to the increased prevalence of overweight/obesity. Successful cancer prevention requires government policies and actions [2]. Risk communication should not be viewed as a substitute for regulation or action but should instead encourage such actions [39].

### Strengths and limitations

We followed the Reflexive Thematic Analysis Reporting Guidelines [42] to enhance the quality of this study. Reflexivity is crucial in RTA. I (ÅG), therefore, reflected on my pre-understanding and positionality in relation to the study, as well as the risk of introducing weight bias into the research. Although I, as a researcher, have theoretical knowledge about the potential harms of risk information, I have never struggled with overweight or experienced discrimination. During the interviews, I strived to adopt a humble, non-judgmental, empathetic approach, where the participants’ experiences are validated [43]. I consulted a patient organisation before recruitment to discuss considerations such as the choice of words for overweight/obesity. We also had repeated discussions within the research group about our reflections.

We made an active choice not to ask for or assess participants’ weight during recruitment or data collection, since that would risk introducing weight stigma [43]. Instead, we allowed individuals to self-assess whether they met the inclusion criteria of having overweight or obesity. We also presented multiple ways to conduct the interviews, including telephone interviews, which have the benefit of removing the influence of visual body size differences between the researcher and participant, thereby reducing the researcher-participant power imbalance present in face-to-face interviews [43]. Despite these considerations, we struggled with recruitment. Only a few participants signed up, and two withdrew from participation, which may be a sign of stigma [43]. Using the concept of information power, we assessed that the amount of data collected was sufficient to answer the research question. The data was rich and provided in-depth insights into the participants’ lived experiences. However, their diverse life experiences, e.g., experiences with health issues and healthcare visits, indicate that a couple more interviews could have further enriched the descriptions.

## Conclusion

Participants experienced that risk information, when presented as simplified associations between obesity and cancer, felt personally relevant but difficult to act upon, and therefore burdensome to bear. Consequently, the information risks failing to prevent cancer and may instead cause harm to the people it purports to benefit. Participants suggested several ways to improve the communication and effectiveness of the information, such as introducing multiple ways to reduce cancer risk beyond weight reduction and raising public awareness about the causes of obesity. Risk information efforts, directed at the public or patients, require better ethical considerations of their benefits and potential harms. Furthermore, risk communicators should act with compassion and a sense of responsibility.

## Electronic supplementary material

Below is the link to the electronic supplementary material.

Supplementary Material 1

## Data Availability

Due to the formulations of the informed consent form, study data cannot be made publicly available. Data are however available from the corresponding author upon reasonable request subject to ethical permissions and participant consent.

## Abbreviations

BC: Breast cancer
CRC: Colorectal cancer
ECAC: European Code Against Cancer
HPC: Health care professionals
RTA: Reflective thematic analysis
