# Correction: Perceptions of cancer risk communication in individuals with overweight or obesity– a qualitative interview study

**DOI:** 10.1186/s12889-025-23788-9

**Published:** 2025-07-12

**Authors:** Åsa Grauman, Erica Sundell, Jessica Nihlén Fahlquist, Mariann Hedström

**Affiliations:** 1 Centre for Research Ethics and Bioethics, Uppsala University, Box 564, Uppsala, SE-751 22 Sweden; 2 Department of Public Health and Caring Sciences, Uppsala University, Box 564, Uppsala, SE- 751 22 Sweden

**Correction: BMC Public Health 25**,** 1900 (2025)**

**https://doi.org/10.1186/s12889-025-23056-w**

Following publication of the original article [1], the authors identified an error to the author names. The given name and family name were erroneously transposed for all authors.

The incorrect author names are:

Grauman Åsa

Sundell Erica

Nihlén Fahlquist Jessica

Hedström Mariann

The correct author names are:

Åsa Grauman

Erica Sundell

Jessica Nihlén Fahlquist

Mariann Hedström

The author group has been updated above and the original article [1] has been corrected.
