# Supplementary material for: Perceptions of cancer risk communication in individuals with overweight or obesity– a qualitative interview study
Source: BMC Public Health. 2025 May 23;25:1900. doi: 10.1186/s12889-025-23056-w (PMC12101019; doi:10.1186/s12889-025-23056-w)
Supplement: Supplementary file 1 — Supplementary Material 1 [file 12889_2025_23056_MOESM1_ESM.docx]

**Supplementary file: Interview guide translated into English.**

[Translation assisted by DEEPL Translator]

Breast cancer and bowel cancer are two of the most common cancers in Sweden. Do you have any idea what causes these cancers?

There are many different factors that can influence the risk of developing these cancers. Apart from bad luck, the risk is affected by factors that the individual cannot influence, such as genetics, but also by factors that can be influenced, such as an individual's lifestyle and weight (smoking, alcohol consumption, physical activity, eating habits and overweight and obesity).

Do you remember coming across information that being overweight or obese increases the risk of certain cancers such as breast cancer and colorectal cancer?

(If Yes) Can you tell us about that occasion? (in the media, news, newspaper, TV, social media, or received such information directly addressed to you as a person?) What was the situation? Was it addressed to you personally or to people in general?

How did you react to that information? What did you think when you read/heard about it? What emotions did it evoke in you? Did it make you want to act in any way?

Have you also been told about it in a face-to-face meeting, e.g. in a healthcare setting? How did you react to that information? What did you think when you read/heard about it? What feelings did it evoke in you? Did it make you want to act in any way?

How do you feel about the way risk information about how overweight and obesity as a risk factor for cancer was conveyed back then? What was good? What was bad?

(If No)

Imagine reading or hearing on the radio that researchers have concluded that being overweight or obese increases the risk of breast cancer and colorectal cancer. How would you react to this information? What would you think if you read/heard about it? What feelings would it evoke in you? Would it make you want to act in any way?

If you imagine your doctor raising it during a healthcare visit, how would you react to that information? What would you think about it? What feelings would it evoke in you? Would it make you want to act in any way?

Is this type of information useful to you? In what way? How do you think information about how overweight and obesity increase the risk of cancer should be communicated to be useful? Should it contain anything specific to be useful and meaningful?

Does this type of information affect you negatively in any way? How? can you elaborate?

Do you feel that this type of information can make you feel responsibility for your health? How does it make you feel?

Could it have other negative consequences for people with obesity? (e.g. in terms of how other people/society think about, treat people with obesity?

Do you think media coverage of the link between obesity and cancer could affect how people in the general public think about people with obesity? Or how they treat people who are overweight or obese?

What is important to consider when communicating risk information about how lifestyle behaviours affect breast/colon cancer risk?

Are there people or professional groups that you think are better suited than others to provide such information? From whom you would prefer receive information?

Are there times that are better suited for such information?

Are there times that are less suitable for such information?

**Closing questions:**

Those are all the questions I had. [Give a short summary of what was said]. Do you think it's an okay summary or is there anything I misunderstood or should be added?

Thank you for your participation!

**Probes:**

- Can you tell me more about it?

- Can you think of other aspects?

- What do you think/feel about it? Is it good or bad?

- What feelings/reactions does it evoke?
